# Challenges and Obstacles in Applying Therapeutical Indications Formulated in Molecular Tumor Boards

**DOI:** 10.3390/cancers14133193

**Published:** 2022-06-29

**Authors:** Edoardo Crimini, Matteo Repetto, Paolo Tarantino, Liliana Ascione, Gabriele Antonarelli, Elena Guerini Rocco, Massimo Barberis, Luca Mazzarella, Giuseppe Curigliano

**Affiliations:** 1 Division of Early Drug Development, European Institute of Oncology, IRCCS, 20141 Milan, Italy; edoardo.crimini@ieo.it (E.C.); matteo.repetto@ieo.it (M.R.); liliana.ascione@ieo.it (L.A.); gabriele.antonarelli@ieo.it (G.A.); luca.mazzarella@ieo.it (L.M.); giuseppe.curigliano@ieo.it (G.C.); 2 Department of Oncology and Hematology (DIPO), University of Milan, 20122 Milan, Italy; elena.guerinirocco@ieo.it; 3 Division of Pathology, IEO, European Institute of Oncology IRCCS, 20141 Milan, Italy; massimo.barberis@ieo.it

**Keywords:** molecular tumor board, precision oncology, targeted therapy, drug accessibility, cancer, oncology

## Abstract

**Simple Summary:**

Precision oncology is gaining a great deal of interest as it aims to administer the right drug to the right patient at the right moment, overcoming the classical paradigm of “one drug fits all”. Molecular tumor boards (MTBs) are multidisciplinary groups instituted to formulate the most up-to-date therapeutical or diagnostic indication for cancer patients performing genomic analyses. Unfortunately, MTBs must face many issues in applying the indications formulated and only a small percentage of patients effectively receives the targeted treatment. In this review, we aim to describe the most common issues that impede the implementation of MTB indications and the solutions that have been developed.

**Abstract:**

Considering the rapid improvement of cancer drugs’ efficacy and the discovery of new molecular targets, the formulation of therapeutical indications based on the multidisciplinary approach of MTB is becoming increasingly important for attributing the correct salience to the targets identified in a single patient. Nevertheless, one of the biggest stumbling blocks faced by MTBs is not the bare indication, but its implementation in the clinical practice. Indeed, administering the drug suggested by MTB deals with some relevant difficulties: the economical affordability and geographical accessibility represent some of the major limits in the patient’s view, while bureaucracy and regulatory procedures are often a disincentive for the physicians. In this review, we explore the current literature reporting MTB experiences and precision medicine clinical trials, focusing on the challenges that authors face in applying their therapeutical indications. Furthermore, we analyze and discuss some of the solutions devised to overcome these difficulties to support the MTBs in finding the most suitable solution for their specific situation. In conclusion, we strongly encourage regulatory agencies and pharmaceutical companies to develop effective strategies with medical centers implementing MTBs to facilitate access to innovative drugs and thereby allow broader therapeutical opportunities to patients.

## 1. Introduction

Advancing oncology research to ultimately introduce novel effective treatments is still an urgent need, since cancer is among the leading causes of death worldwide. In this setting, a key role has emerged in recent years for precision medicine, namely the customization of treatment to a patient’s and a tumor’s characteristics. Precision medicine approaches are significantly expanding their applications in oncology, with next generation sequencing (NGS) being a diagnostic tool that has become part of oncologists’ armamentarium to better describe a specific tumor’s characteristics and to define paths towards personalized cancer therapies. In depth, molecular tumor profiling helps in recognizing druggable gene alterations and identifying those eventually conferring resistance to anticancer therapies or a predisposition to treatment-related toxicities [1]. Nevertheless, the interpretation of NGS results and the formulation of the most appropriate therapeutical indication according to the most recent evidence requires the combined effort of different healthcare professionals, as the treating oncologist must manage a variable spectrum of molecular tests, provided by different commercial services or academical institutions [2]. For instance, Foundation One CDx is a largely used NGS panel including 324 cancer-related genes to be performed on a tissue sample [3]; the same company also developed a liquid biopsy test, the Foundation One Liquid CDx [4]. Caris Molecular Intelligence is another comprehensive genomic profiling service that includes a large variety of cancer genes and other histotype-specific biomarkers and demonstrated its validity in a clinical setting [5]. 

To face the complexity of precision oncology, molecular tumor boards (MTBs) have been founded and are spreading among different institutions worldwide [6]. MTBs are multidisciplinary meetings during which multiple professionals (i.e., medical oncologists, pathologists, geneticists, pharmacologists, molecular biologists, bio-informaticians, etc.) share their expertise to discuss NGS reports and integrate these data with a patient’s clinical history. Their main purpose is to ultimately identify available therapies matched to the single patient’s tumor profile and to address other management needs (i.e., the need for genetic consultation or further testing) [7].

During MTBs, all molecular findings are discussed and classified according to the most up-to-date evidence to distinguish benign or neutral alterations from pathogenic ones, even confirmed or likely. For this purpose, different online databases are available, including ClinVar [8], Cosmic [9], or OncoKB [10]. In particular, OncoKB is the first somatic mutation archive approved by the Food and Drug Administration (FDA) [11]. 

In addition, due to the broader role of immunotherapy in cancer treatment, other relevant aspects to be considered are signatures, such as tumor mutational burden (TMB), identified by NGS, and the presence of mismatch repair deficiency (dMMR)/microsatellite instability (MSI-H), evaluated both by NGS or by immunohistochemistry (IHC). In fact, for tumors with MSI-H/dMMR and/or with a TMB > 10 Mutations/Megabase, immunotherapy with PD1-inhibitors represents a therapeutical choice, according to the FDA agnostic approval of pembrolizumab or dostarlimab [12,13]. 

Once all the molecular data have been annotated and integrated with the patient’s oncological and non-oncological history, it is possible to frame a therapeutic recommendation by classifying potential targets in relation to the clinical evidence of its utility, according to specific scales (i.e., the ESMO (European Society of Medical Oncology) Scale for Clinical Actionability of molecular Targets, ESCAT [14]; or the joint consensus recommendation, JCR [15]).

The MTB letter output is usually a report signed by all the different professionals attending the meeting and it should contain information regarding:

1. The collection of the relevant parties’ informed consent;
2. The patient’s clinical data (comorbidities, ongoing therapies, and the presence of target lesions according to RECIST 1.1 criteria) [16];
3. NGS data and all other molecular findings (i.e., polymerase chain reaction, PCR; fluorescent in situ hybridization, FISH; IHC);
4. The databases utilized to annotate the pathogenicity of each molecular alteration found;
5. The need for genetic counselling;
6. The final recommendations with the relative levels of evidence;
7. The availability of clinical trials in which the patient can be enrolled and the information regarding enrolling centers or whether there is any open expanded access program;
8. The relevant bibliography utilized.

Of note, the MTB report is not yet structured nor standardized across various institutions, as with some pathology reports [7]. So far, the most investigated cancers in MTBs are represented by rare tumors—with sarcomas being the most common histotype [7]. In general, MTBs are a resource that is commonly employed in an advanced setting: when patients generally have no further standard of care therapies available. Moreover, among the most common alterations discussed in MTBs are *TP53*, *KRAS*, and *PIK3CA* gene mutations [17,18]. 

Although the results from prospective trials are still scarce, with the only randomized controlled phase II trial (SHIVA) showing no added benefit from MTB-based molecularly targeted agents [19], data from a systematic review by Larson et al. confirmed that MTB-recommended therapies resulted in slightly improved clinical outcomes, although with great variability in the response rates [20]. Single institutions are evaluating MTBs’ impact based on their experience; however, the real value of MTBs should be weighted not only at the patient’s or single institution’s level but also considering the entire scientific community.

## 2. Issues in Applying MTB Output

One of the most important questions after the MTB discussion is to find the best way to apply the formulated indications. Firstly, the global MTB turnaround time, defined as the time necessary for genomic testing and for MTB output, should be maintained within a reasonable period (Luchini et al. proposed 28 days [7]) to avoid the worsening of clinical conditions that could impede the patient’s enrollment. Furthermore, if the turnaround time is so elevated that the patient cannot await the therapeutical indication without starting a new line of therapy, the MTB output could consequently be outdated in its application in the subsequent lines. According to the systematic review of Luchini et al., the mean turnaround time for MTBs was 38.4 days for the analyzed studies, with an elevated variability among them [7]. Nevertheless, the global MTB turnaround time strictly depends upon the turnaround time of the molecular testing, which is progressively shortening. For instance, Foundation One, which is one of the most employed commercial NGS services, reduced its turnaround time from the 15 days (range 7–30 days) reported in 2014 to the guaranteed less than 12 days since the specimen’s arrival for the Foundation One CDx test [3,21]. Hopefully, this time will reduce increasingly further as NGS testing spreads in clinical practice. Moreover, MTB-specific turnaround time can be shortened by implementing new approaches instead of the classical in-person meetings at fixed dates. For instance, a virtual MTB showed an excellent turnaround time from the data entry to the MTB output of 4 days, improved within 4 years of the project’s development [22]. Considering these data, it is not a utopian suggestion that in the near future a less than 15-day turnaround time could be reached. 

Apart from the turnaround time, the economical aspect very often plays a central role in the application of the therapeutical indication of the MTB. In fact, not infrequently, the indication is an off-label utilization of drugs approved in different cancer types or not approved by the regulatory agency of the patient’s country. For instance, 74% of the indications formulated in the Comprehensive Cancer Center of Freiburg MTB were off-label treatments [23], and many other cancer centers reported their experiences with many indications being off-label [24,25]. Nevertheless, according to the ESMO recommendations for the use of NGS, the ESMO “recommends using off-label drugs matched to genomics only if an access program and a procedure of decision have been developed at the national or regional level” [26]. 

Considering that targeted therapies are usually innovative molecules with elevated prices, only few patients can afford to pay out of their own pockets. A prospective cost study of the MOSCATO trial showed that the global cost of the molecular-guided therapy was €31,269 per patient, with anticancer drugs concurring for 54% of amount [27]. Therefore, this kind of indication very often remains unapplied in the absence of a clinical trial, an expanded access, or a compassionate use program through which the patient can obtain the drug. 

Nevertheless, even when a clinical trial could be suitable for the patient, geographical, economic, or social difficulties can impede the enrolment. 

For example, clinical trials are usually not distributed uniformly on the territory, leading to difficulties in the enrolment of cancer patients that do not have a specific trial near their domicile [28,29,30]. This problem arises from underlying economic, social, and cultural disparities between patients, allowing only highly motivated patients to travel, and with more resources required to be enrolled within a clinical trial. According to a 2007 survey, only 37% of cancer patients would be willing to travel to be enrolled in a clinical trial in the USA [31]. Further evidence highlighting the disparities present in access to clinical trials can be derived from an Indian study showing a significant inter-state difference [30].

## 3. Role of Pharmaceutical Companies 

Pharmaceutical companies play a key and progressively more relevant role in directing pharmacological research and subsequently in providing the chance to receive a specific treatment matched with a patient’s molecular alteration. This role is highlighted by the progressive increase of cancer drugs’ approval in the last decades, passing from the 41 drugs approved in the 1990–1999 decade to the 105 approved between 2010 and 2019 [32]. Concomitantly, the drug-related costs for health systems and for patients increased [33]. In fact, a retrospective study highlighted how the annual revenue from cancer drugs of 10 big pharmaceutical companies increased by around 70% ($55.8 billion to $95.1 billion) from 2010 to 2019, while non-cancer-related drugs’ revenue decreased by 18% [34]. Overall, this pharma-driven research model risks side-lining clinicians and their specific relationships with the patients and nourishes a vicious circle that may progressively set aside the academic and spontaneous research into the economic logic underlying drug development.

In this framework, conducting a clinical trial is becoming increasingly expensive and the sponsorship of pharmaceutical companies is often needed to effectively develop new drugs that could have an impact on the outcome of cancer patients. A study evaluating the estimated costs of pivotal clinical trials leading to the FDA-approval of 101 new therapeutic agents from 2015 to 2017 showed an estimated median cost per molecule of $48 million, while the estimated per patient cost was $41.413 [35]. On the other side, clinical trials have the double advantage of allowing an early access to innovative drugs and the acquirement of high-quality prospective data on safety and efficacy [36]. From the patient’s point of view, participating in a clinical trial, especially in the early phase, means being followed with more attention than a patient receiving an already approved therapy in clinical practice [37]. The other side of the coin of these advantages for the enrolled patients are the progressively stringent inclusion and exclusion criteria intended to select the patients in order to analyze the nearest-possible ideal population, the travelling costs for patients (due to the frequent hospital visits), and the frequent need to undergo invasive procedures for study purposes (e.g., tumor biopsies) [36]. 

Overall, clinical trials, when available, are a good opportunity for the patients discussed in MTBs to receive targeted therapy. However, only less than 3% of cancer patients are actually enrolled in a clinical trial [38]. For patients that are not eligible, other pathways for accessing experimental or not yet approved drugs have been developed, for example, the Expanded Access Programs (EAP) or the less bureaucratic pathway created with the Right-To-Try Act in the United States of America [39]. However, pharmaceutical companies hold the ultimate decision on the requests of the drug, potentially dramatically impacting the outcome of the single patient outside clinical trials [39].

Pharmaceutical companies are also deeply involved in academic research, especially when considering basket or platform studies of precision medicine, as the drugs must be granted for the trial. It is logical that pharmaceutical companies are more willing to concede the utilization of molecules that have already demonstrated strongly positive results and whose development and approval cannot be endangered by the eventually negative results of the trial, or on the contrary, molecules that failed in their pivotal trials and can only benefit by a positive result to be repurposed.

## 4. Overview of MTB Experiences, Precision Medicine Trials, and Solutions for Increasing Matched Therapies Access

Based on what has been reported in the previous paragraphs, it is clear that the role of the MTB is very thorny and the possible solutions to ease the access to targeted drugs for cancer patients must be developed in the specific regulatory context in which the MTB has to operate. In general, the methods for increasing the percentage of cancer patients harboring an actionable molecular target receiving a molecular-matched therapy can be divided into: (i) screening programs without access to drugs, (ii) platform or umbrella interventional studies in which patients can receive the drug within the trial, and (iii) strategies aimed at maximizing the accrual of clinical trials. Table 1 contains an overview of the literature illustrating the most frequent reasons preventing access to the indicated targeted therapy.

### 4.1. Institutional MTB Experiences 

Here we describe the most important studies reporting MTB experiences. We have selected articles including at least 100 patients and indicating the modalities through which patients received the treatment indicated by the MTB. The percentage of patients receiving matched therapies refers to the patients that received a therapeutical indication or, if not specified, the number of patients with actionable mutations.

The MTB of the Institut Curie discussed 736 cancer patients from 4 October 2014 to 31 October 2017, among which 442 performed molecular analyses [18]. At least one actionable alteration was identified in 207 (47%) patients, among which 45 (21%) of them were included in a clinical trial, while 7 (3%) received the drug as an off-label indication, while no info was available concerning other treatment access modalities [18]. The most common reasons for the failed inclusion in clinical trials were patient death (34 patients), a lack of trials (30 patients), the patient was lost to the follow-up (25 patients), ineligibility (19 patients), other treatments were made available (19 patients), matched drugs were already received (7 patients), or patient refusal (5) [18]. 

The Sidney Kimmel Comprehensive Cancer Center MTB in its starting three years since October 2013 discussed 155 patients: 132 (85%) had actionable alterations and in 37 (24%) patients off-label therapies were recommended [40]. Twenty-nine (78%) patients were treated according to the MTB indication, including 13 treated in clinical trials, 11 that received off-label drugs, and 5 that received FDA approved therapies. A total of 46 patients, instead, received non-matched therapies, approved or in clinical trials, of which 19 did not have clinical trials available, 9 showed a worsening performance status (PS) that did not allow for other treatments, and 19 were lost to follow-up [40].

One-hundred patients with rare or refractory tumors were sequenced in a prospective trial from April 2013 to December 2013 at the Rutgers Cancer Institute of New Jersey and discussed at MTB [41]. A total of 87 (87%) patients had actionable alterations, and 31 (36%) received a matched therapy in a clinical trial, off-label use, or FDA approval [41]. A total of 26 patients did not receive the matched treatment due to the lack of availability of a clinical trial or access to an FDA-approved drug, 12 had a rapid deterioration of PS, and 8 were lost to the follow-up [41].

The MTB of the University of Alabama at Birmingham discussed 191 cases, providing indications of molecular testing for 132 patients: 48 (39%) harbored actionable alterations, 15 (31%) received a targeted treatment, 13 received standard treatment, 10 were referred to hospice, and 8 had no follow-up data [42]. The necessity for providing an indication for genomic testing by MTB arose from the unavailability at the institution of the clinical trials in which patients could perform such analyses and the charges for the hospital and patients were excessive, so an agreement with an academic collaborator was established for reimbursing the genomic tests prescribed by MTB [42]. 

The Sarah Cannon Research Institute UK/UCL Genomics Review Board reviewed 895 cases of cancer patients that had performed genomic testing [43]. A total of 76 patients (8.5%) received an approved therapy based on molecular profiling, while 47 (5%) were enrolled in molecular matched clinical trials, and 8 (1%) received target therapy as compassionate use, but the number of patients with actionable mutations and the reasons for not receiving matched therapies were not reported [43].

The Antwerp MTB, in Belgium, discussed 173 cases of advanced cancer patients, indicating for 72 (46%) a targeted therapy: 49 in clinical trials and 23 in expanded access programs or as off-label studies [6]. Unfortunately, the number of patients receiving the targeted therapy was not specified, nor were the reasons for not receiving the targeted treatment [6]. 

### 4.2. Precision Medicine Trials—Screening Programs Reporting Drug Access Data

In a pilot study whose results were published in 2010, 86 of the 106 advanced cancer patients enrolled underwent molecular screening, and actionable molecular alterations were detected in 98% of them [44]. A total of 66 (78%) patients were treated with commercially available drugs, which were suggested by the coordinating center to the treating physician, and 18 of them had a progression-free survival (PFS) of matched therapy/PFS of a previous line of therapy (PFS2/PFS1) ratio > 1.3 [44]. The two reasons reported by the authors for not accessing targeted therapy were the worsening of clinical conditions and patient unwillingness [44]. 

Mi-ONCOSEQ began its activity in 2011 [45]. The study globally enrolled 1138 patients, with 1015 having a successful NGS testing, and all the cases were discussed in an institutional MTB [45]. Among them, 817 (80.5%) harbored an actionable alteration, but only 132 (16.7% of the 817) started a sequencing-directed therapy, with a clinical benefit rate (CBR) of 37.1% [45]. A total of 74 patients received the sequencing-directed therapy in a clinical trial, 43 as an off-label treatment, and 15 as an on-label indication, but the reason why 83.3% of patients did not obtain the targeted therapy was not reported [45].

Between March 2012 and July 2013, 2601 patients were enrolled in the MD Anderson Cancer Center Personalized Cancer Therapy Program and performed a genomic profiling with different small panels, but for 601 patients the analysis failed [46]. A total of 789 patients had an actionable molecular alteration (39%), among which 83 (11%) were enrolled in a genotype-matched trial in the institution [46]. The authors then analyzed the 429 patients with *PIK3CA/AKT1/PTEN/BRAF* mutations, discovering that 199 were no longer treated in the institution (17% did not return after testing, 13% were treated near the domicile, 6% had rapid clinical condition worsening, and 10% for other reasons, usually for disease control), while 96 patients among the other 230 (42%) received a genotype-matched therapy after testing (40 as an off-label or clinical practice use) [46]. 

From March 2012 to July 2014, 1893 patients were enrolled and 1640 tested in Princess Margaret Cancer Center IMPACT/COMPACT trial [47]. The difficult cases were discussed in an institutional MTB and 84 patients (5%) were then treated in a genotype-matched trial [47]. Interestingly, patients who were enrolled in a genotype-matched trial had an objective response rate of 19% versus 9% of the genotype-unmatched trials, but no differences were detected in the overall survival (OS) nor in the time on treatment [47]. The trial investigators tried to increase the accrual in clinical trials via MTB timely discussions, alerts to physicians containing genotype-matched trial reporting, and individual physician summaries of the profiling results, but the major issues impeding the enrolment were mainly geographical accessibility, a lack of trials, and performance status impairment [47]. 

An MSK-IMPACT analysis of 10,336 advanced cancer patients treated at Memorial Sloan Kettering Cancer Center showed that 3793 (36.7%) of them harbored at least one actionable molecular alteration [48]. Considering the first 5009 patients tested, 527 (11%) were enrolled in a molecularly-matched clinical trial in the institution, but the total number of patients receiving a targeted therapy as off- or on-label indication, nor the ones enrolled in clinical trials in other institutions, were reported [48]. The authors enumerate geographical accessibility, patient preferences, a lack of pertinent clinical trials, and the worsening of the clinical condition as the most likely reasons for not receiving the targeted therapy [48]. Interestingly, an automated system (DCMS) that sends the results of genomic testing to an institutional database and signals the eligibility of the patient within a specific trial to the pertinent physician was developed in the institution [49]. This system standardizes and systematically allocates patients to clinical trials, maximizing the enrolment [49].

The CoPPO trial was designed in 2013 to assess the utility of comprehensive genomic profiling to guide the inclusion of cancer patients in phase I trials [50]. A total of 352 patients of the 500 biopsied cancer patients (70%) harbored an actionable alteration, and 101 (20%) received the targeted therapy indicated by the institutional MTB [50]. The rapid worsening of the clinical condition was the most common reason for not receiving the targeted therapy (151 patients out of the 352 with actionable alterations), while for the other 100 there were no available matched clinical trials in the institution [50]. A total of 15 patients (15%) presented CBR from the matched treatment [50].

A clinical trial that aimed to establish the utility of performing whole-exome sequencing (WES) between 2013 and 2014 included 97 patients with advanced cancer, mainly prostate and urothelial neoplasms [25]. A total of 91 (94%) patients harbored actionable mutations and were discussed during a dedicated MTB, but only 5 (5%) obtained the matched therapy in clinical trials or as off-label use [25].

From April 2014 to October 2015, 168 advanced cancer patients were referred to the Indiana University Health Precision Genomics Program, where they underwent molecular screening and were discussed at MTBs [51]. A total of 67 patients were excluded from the final analysis, primarily (40 patients) because no further therapies were received after the molecular screening or because they were lost to the follow-up. Among the remaining 101 patients, 44 received a molecular-matched treatment (44%), while the remaining 57 did not because of lacking actionable alterations, an unspecified inaccessibility to the treatment, or physician choice [51]. Regarding the efficacy results, 43.2% of patients who received matched therapy had a PFS ratio of >1.3 compared to 5.3% of patients receiving non-matched treatment [51].

Another clinical trial conducted at MD Anderson Cancer Center included 500 advanced cancer patients, and 339 had a successful molecular testing performed [52]. A total of 315 (93%) of them harbored potentially actionable molecular alterations, and 188 received a treatment; 122 (36% of the 339) were matched and 66 unmatched, but it was not reported how the treatments were obtained [52]. Among the 134 patients with a successful genomic profiling that did not receive a matched therapy, 79 died before having the possibility to start the treatment, 32 were still on a previous treatment, 8 were lost to follow-up, 4 refused, and 1 required only a watchful-waiting approach [52]. This trial showed that patients with higher matching scores were more likely to obtain clinical benefits from the treatment [52].

Similarly, in the MASTER trial, 362 (32%) out of the 1138 patients affected by rare tumors that received a therapeutical indication during the MTB discussion obtained the suggested drug [53]. Analogously to the precedents, this trial did not provide for specific treatment access, so the drugs were obtained within clinical trials, compassionate uses, or off-label treatments [53]. 

From April 2013 to December 2015, the multicentric WINTHER trial enrolled 303 advanced cancer patients, among which 158 (52%) received a therapeutical indication by the central management committee and 107 (68%) were allocated to the treatment suggested, not necessarily molecularly matched, according to the molecular profiling obtained, with DNA sequencing (69 patients, arm A) or RNA expression (38 patients, arm B) [54]. Globally, 159 drugs were administered, among which 115 were via off-label use, 22 approved on-label, and 22 were investigational compounds [54]. The main reason for not receiving the treatment proposed was clinical deterioration or death, while almost half of the patients did not have an adequate molecular profiling due to inaccessible tumor sites for biopsy, clinical deterioration, or the tumor sample quality [54]. Of note, adding the transcriptomic analysis enabled an increase of the percentage of patients treated from 23% to 35%, even if the number of patients harboring actionable alterations was not specified [54]. This study did not meet its primary endpoint of PFS2/PFS1 > 1.5 in at least 50% of patients [54].

The Investigation of the Profile-Related Evidence Determining Individualized Cancer Therapy (I-PREDICT) trial enrolled 149 patients, who underwent genomic profiling tests and were discussed in a dedicated MTB, with an indication possibly to combination therapies targeting a majority of alterations in each patient [55]. A total of 83 (56%) patients were evaluable for the analysis; while 43 never started a treatment, mainly because of clinical deterioration or death; 14 were treated for less than 10 days for the same reason; and 9 were still awaiting the results of the test [55]. A total of 73 (49%) patients out of the 83 treated received a molecular matched treatment, 28 with a matching score > 50% [55]. The remaining 10, despite 9 of them having a potentially actionable molecular profile, did not receive the matched therapy because of the treating oncologist’s choice (36.4%), patient preference (36.4%), taking part into another clinical trial (18.2%), or concerns about drug toxicities (9.1%). A total of 75% of patients with matching score > 50% had a PFS2/PFS1 ratio > 1.3, versus 36.6% where the matching score was <50% [55]. Overall, this trial had a considerable matching rate of 49%, which was achieved with shrewdness: I) cases of patients that needed to rapidly start a therapy were discussed in extraordinary MTBs as soon as the genomic report was available; II) employment of a medication acquisition specialist and clinical trials coordinator to guarantee timely access to drugs [55]. Targeted therapies were administered according to insurance coverage for off-label agents and the availability of clinical trials, as per the United States clinical practice [55].

The TARGET trial enrolled 100 advanced cancer patients, aiming to compare the reliability of liquid biopsy to tissue sample NGS in guiding the therapeutical choice [56]. All the patients were reviewed in a dedicated MTB, which formulated a therapeutical indication in 41 (41%) patients with actionable alterations, 11 (27%) received the matched treatment, 17 a non-matched, and 13 did not have any trial available or deteriorated rapidly [56]. Interestingly, this study reports that the integration of clinical and genomic data is one of the major challenges for producing an MTB output useful for the patient, so the digital tool eTARGET, integrating all the patients’ characteristics, was developed and guaranteed a rapid and comprehensive evaluation of eligibility for the clinical trials [56].

The GOZILA trial, a Japanese molecular screening program employing circulating tumor DNA (ctDNA), enrolled 1687 patients affected by gastrointestinal cancer [57]. Interestingly, ctDNA genotyping reduced the time necessary to enroll the patient within a clinical trial (11 vs. 33 days, *p* < 0.0001) and increased the percentage of enrolled patients (9.5% vs. 4.1%, *p* < 0.0001), when compared to tissue genomic profiling [57]. 

Considering breast cancer, AURORA was a molecular screening initiative that aimed to characterize the molecular features and differences between primary and metastatic cancer sites in the same patient [58]. A total of 51% of the patients harbored an ESCAT tier I or II alteration (36% excluding ERBB2 alterations), but only 7% received a genomic-matched therapy [58]. 

### 4.3. Precision Medicine Trials—Basket and Platform Trials

The inclusion of MTBs in the precision medicine platform trials is an interesting solution to guaranteeing access to targeted therapies. For example, SHIVA was a prospective platform trial that started in 2012 and included patients with multiple advanced solid tumors [19]. Patients performed a large-scale molecular testing and received one of the eleven targeted therapies if an alteration in the hormone receptor, *PI3K/AKT/mTOR* or *RAF/MEK* pathways was detected [19]. Despite the multiple criticalities [20], the small number of targeted therapies available, and the negative results, a remarkable fact from the data is that 40% of the included patients received the molecular-matched therapy [19]. 

The MOSCATO trial started enrolment in 2011 and each patient underwent a high-throughput molecular screening [59]. A total of 411 patients out of the 843 with a successful NGS analysis (49%) had an actionable molecular alteration, while 199 (24%) received a targeted therapy according to the indications of a study-dedicated MTB [59]. The most frequent reason for not receiving the targeted therapy was a rapid clinical deterioration (64 patients), then inclusion in another trial (45 patients), and exclusion criteria (21 patients) [59]. The primary endpoint of the trial, the PFS2/PFS1 ratio, was met, as 33% of patients had PFS2/PFS1 > 1.3 [59].

In 2015, the NCI-MATCH platform trial started its activity, allocating patients in 24 subprotocols with 17 different targeted therapies according to the eventual molecular alteration identified in the screening phase [60]. The trial started with a disappointing assignment rate to the initial 10 subprotocols of 5.1%, but it increased to 25.3% when all the 24 subprotocols were active [60]. The trial did not assign to treatment patients for whose molecular alterations approved drugs were available, so it is difficult to calculate the real percentage of patients who received a targeted therapy [60]. 

The ProfiLER trial enrolled 2579 advanced cancer patients, among which 1980 obtained a conclusive molecular profiling and 1032 (52%) had actionable alterations. Of them, 699 (68%) received molecular-matched therapy recommendation by the MTB. However, only 163 (23%) actually received the suggested regimen, with 28% of drugs delivered by off-label use [61]. The primary reasons for the low adherence to MTB recommendation were drug or clinical trial unavailability and early death, but an accurate account was not conducted [61]. The authors believe that the long timeframe (86 days) for the genomic testing results is at least partially responsible for these results [61]. An update of this trial regarding only gynecological cancer was also published and showed similar data (42% of actionable mutations and 12% received a matched therapy) [62].

The Biscay platform trial screened 391 patients affected by urothelial cancer to identify alterations in the *FGFR* or *mTOR/PI3K* pathways or HRD in order to administrate durvalumab in combination with FGFR inhibitors, mTOR inhibitors, or PARP inhibitors, respectively [63]. Even if combination therapy did not show an increase in progression-free survival (PFS) over durvalumab and AZD4547 monotherapy, 135 patients (34%) were allocated to the treatment [63].

MyPathway is an ongoing phase IIa open-label multiple basket trial which employs Trastuzumab, Pertuzumab, Erlotinib, Vemurafenib, Cobimetinib, Vismodegib, Alectinib, and Atezolizumab in advanced cancer patients harboring alterations in *ERBB2*, *EGFR*, *BRAF*, *Hedgehog*, *ALK* pathways, or high TMB [64]. We report it for completeness reasons, but this study does not perform a molecular screening, so alterations must already beknown before the enrolment and all the patients whose alterations are centrally confirmed start the treatment; therefore, an MTB discussion has not been held [64]. The results of different cohorts have been published, but their reporting is out the scope of this review [64,65,66,67,68].

Analogously to MyPathway, the large ongoing Targeted Agent and Profiling Utilization Registry (TAPUR) study aims to find signals of efficacy of the commercially available targeted therapies in patients affected by advanced cancer with already known potentially actionable mutations [69]. The results of several cohorts have been already reported [70,71,72,73]. 

On the other hand, the VIKTORY umbrella trial enrolled 772 advanced gastric cancer patients, and 105 out of the 715 (20.6%) that had a successful targeted sequencing received a molecular matched therapy [74]. 

Recently, the results of the K-MASTER protocol, a pan-cancer precision medicine Korean program, were published [75]. The protocol included 4028 East Asian patients with advanced solid tumors that underwent NGS analysis through one of three oncology-based sequencing panels [75]. Based on the detected molecular alterations, 440 (10% of the total screened) patients have been enrolled so far in 20 different clinical trials, but the complete results are still being awaited [75]. 

The Drug Rediscovery Protocol (DRUP), developed in the Netherlands, is one of the most important programs addressing the issue of accessing the targeted therapy beyond their approved indications by FDA and EMA [76]. The program, still ongoing, recruits advanced cancer patients with no approved treatment options who underwent molecular tumor profiling that showed actionable molecular alterations [76]. A centralized MTB supports the therapeutical indication. The receipt of the targeted therapies in this trial eases the access to the drugs, overcoming reimbursement issues and allowing for data collection, thereby generating knowledge to guide future MTB indications [76]. In fact, these therapies could have been received only under off-label indications outside of the trial, so the development of a personalized reimbursement scheme is of absolute relevance in DRUP protocol: the first stage of each cohort enrolls 8 patients and if at least 1 experiences clinical benefits, the cohort proceeds to stage II, which includes 16 patients with a clinical benefit-rate cutoff of 5 patients for accessing stage III [77]. In the first two stages, the drug is considered an investigational product and is provided for free by pharmaceutical companies, as for the first 16 weeks of each patient in stage III [77]. After 16 weeks, if a clinical benefit is obtained and the effectiveness is proven for the single patient, the drug is reimbursed by the payers as an approved drug [77]. This model enables the sharing of risks and costs between the drug manufacturers and the National Health Care Institute [77]. Interestingly, a recent paper reported the result of the rare tumors included in the study, also updating the total number of patients enrolled [78]. Globally, 500 out of the 1065 (44%) cancer patients submitted to be evaluated for a targeted treatment in the trial received the proposed therapy, with 33% of CBR [78]. 

In the last few years, liquid biopsy has expanded its applications, due to the easier sample collection and the capability of capturing a more comprehensive portrait of all the metastatic sites, thus mitigating the impact of tumor heterogeneity on the results [79]. The PlasmaMATCH platform trial allocated breast cancer patients in four different cohorts according to *ESR1*, *ERBB2*, *AKT1*, and *PTEN* mutations and estrogen-receptor status [80]. A total of 131 (12%) patients received a molecular matched treatment out of the 1051 patients enrolled [80]. Of course, the single histology and the small number of targets included in the study cannot allow one to draw conclusions about the goodness of this approach in the context of applying the indication of the MTBs [80]. 

**Table 1:** Overview of the literature illustrating the most frequent reasons preventing access to the targeted therapies indicated by MTBs. NA: Not Available; TT: targeted therapy.

Institution or Trial Name	Type	Study Period	Total Number of Patients (Actionable Alterations)	Patients Who Received Targeted Therapy	Reported Issues in Applying MTB Indications	Proposed Solutions
Institut Curie [18]	Retrospective—MTB experience	2014–2017	736 (207)	52	Deterioration of clinical conditions; lack of clinical trials; patient’s refusal	-
Sidney Kimmel CCC [40]	Retrospective—MTB experience	2013–2016	155 (132)	29	Lack of clinical trials; deterioration of clinical conditions	-
Rutgers Cancer Institute [41]	Prospective—MTB experience	2013	100 (87)	31	Lack of clinical trials; Deterioration of clinical conditions	-
Alabama University Birmingham [42]	Retrospective—MTB experience	2013–2016	191 (48)	15	Standard treatment preferred; deterioration of clinical conditions	Agreement for the reimbursement of genomic testing prescribed by MTB
Sarah Cannon Research Institute [43]	Prospective—MTB experience	2014–2018	895 (NA)	76	NA	-
Antwerp University Hospital [6]	Retrospective—MTB experience	2013–2017	173 (72)	NA	NA	-
SCRI-CA-001 (NCT00530192) [44]	Prospective -molecular screening	2006–2009	106 (85)	66	Deterioration of clinical conditions; patient’s refusal	-
Mi-ONCOSEQ [45]	Prospective -molecular screening	2011	1138 (817)	132	NA	-
MD Anderson Cancer Center Personalized Cancer Therapy Program [46]	Prospective -molecular screening	2012–2013	2000 (789)	123 (83 + 40 reported in the article)	Deterioration of clinical conditions; geographical accessibility; patient’s refusal; no need for another treatment	-
Princess Margaret Cancer Center IMPACT/COMPACT [47]	Prospective -molecular screening	2012–2014	1893 (NA)	84	Deterioration of clinical conditions; geographical accessibility; Lack of Clinical trials	MTB timely discussions; alerts containing genotype-matched trials; individual summaries of profiling results
Memorial Sloan Kettering Cancer Center [48]	Prospective -molecular screening	2014–2016	5009 (1838—derived)	527 (only clinical trials in MSKCC)	Deterioration of clinical conditions; geographical accessibility; Clinical trials lacking; patient’s refusal	Automated system (DCMS) sending the results of genomic testing to an institutional database and signaling the eligibility of the patient to the pertinent physician
CoPPO [50]	Prospective -molecular screening	2013–2017	500 (352)	101	Deterioration of clinical conditions; Clinical trials lacking	-
Western Regional Medical Center [25]	Prospective -molecular screening	2013–2014	97 (91)	5	NA	-
Indiana University Health Precision Genomics Program [51]	Prospective -molecular screening	2014–2015	168 (NA)	44	Deterioration of clinical conditions; inaccessibility to treatment (unspecified); physician choice	-
MD Anderson Cancer Center [52]	Prospective -molecular screening	2012—unspecified	500 (315—derived)	122	Deterioration of clinical conditions; no need for another treatment; patient’s refusal	-
MASTER [53]	Prospective -molecular screening	2012–2018	(1138)	362	NA	-
WINTHER [54]	Prospective -molecular screening	2013–2015	303 (NA)	107 (treated patients, not specified how many TT)	Deterioration of clinical conditions; no need for another treatment; patient’s refusal	Transcriptomic analysis increased the percentage of treated patients from 23% to 35%
I-PREDICT [55]	Prospective -molecular screening	2015—Unspecified	149 (83)	73	Physician choice;Patient’s refusal; drug toxicity concern.	Timely MTB discussion; employment of a medication acquisition specialist and clinical trials coordinator; Indication possibly to combination therapies targeting a majority of alterations in each patient
TARGET [56]	Prospective -molecular screening	2015—Unspecified	100 (41)	11	Deterioration of clinical conditions; physician choice; lack of clinical trials	Digital tool eTARGET integrating clinical and genomic data
GOZILA [57]	Prospective -molecular screening	NA	1687 (632)	60	NA	Liquid biopsy to shorten analysis time
SHIVA [19]	Prospective—platform	2012–2014	741 (293)	99 (randomized: 96 in control group)	Randomization criteria not met; Deterioration of clinical conditions; patient’s refusal.	-
MOSCATO [59]	Prospective—platform	2011–2016	1035 (411)	199	Deterioration of clinical conditions; physician choice; lack of clinical trials; patient’s refusal.	-
NCI-MATCH [60]	Prospective—platform	2015 (before interim analysis)	795 (56)	33 (only within the trial)	NA	NCI-designed computational platform (MATCHBOX)
ProfiLER [62]	Prospective—platform	2013–2017	2579 (699)	163	Deterioration of clinical conditions (long turnaround time); inaccessibility to treatment (unspecified): no accurate accounting for reasons for not initiating TT was carried out	-
K-MASTER [75]	Prospective—platform	2017—ongoing	4028 (1156—derived)	440	NA	Dynamic precision oncology clinical trials design
DRUP [78]	Prospective—drug access program	2016—ongoing	1065 (NA)	500	NA	Personalized reimbursement model

## 5. Discussion and Recommendations 

Overall, our overview of MTB experiences highlights the difficulty of estimating how many cancer patients really receive the proposed targeted therapy and the proportion of patients actually benefitting from performing an MTB-driven therapeutic indication. The main reason is the lack of structured, centralized, and homogeneous data collection generating real world data, that is also necessary to define, outside the well-established indications, which alterations are really actionable in order to learn from the prior clinical experiences. 

Furthermore, the dramatic heterogeneity of actionable alteration detection among precision medicine studies relies on numerous factors, such as the genomic test performed, the number of genes contained in the panel, the sample quality, the interpretation of the detected variants according to the available evidence, the eventual co-occurrence of resistance mutations, and the drugs available. With this in mind, we strongly encourage the development of a structured report in which the alteration–drug matching is explicated with the associated evidence level to allow for a comparison between different studies. A huge effort is being conducted by oncological institutions such as ESMO to create frameworks such as ESCAT and MCBS in order to help clinicians to homogeneously interpretate clinical trial and genomic test results, and they should be transversely implemented [14,81]. Moreover, it would be useful to track the indications formulated in each MTB to assess their concordance and consistency across different institutions to compare the performance of each approach. 

Globally, the main issues in applying MTB indications highlighted by the authors in the majority of the studies are recurrent and attributable to:

- A long timeframe for genomic testing and/or the MTB output, which increases the risk of clinical deterioration of patients. Henceforth, it is necessary to minimize the turnaround time from the test prescription and the treatment start. Moreover, many authors call for performing these analyses earlier in the patient’s disease course. Nevertheless, the introduction of new technologies in clinical practice, such as liquid biopsy, is facilitating the application of MTB indications. Molecular profiling studies employing this technique (e.g., TARGET, GOZILA) have shown increased percentages of patients receiving targeted therapies and enrolled in clinical trials [56,57]. In fact, the improved manageability and the reduced time for the test results, along with the possibility to have the test performed even without tumor tissue available, could help to rapidly administrate the targeted therapy while avoiding clinical condition deterioration or the necessity of starting another treatment in the meantime.
- Drug accessibility, also in the frame of a clinical trial, a lack of clinical trials, geographical accessibility, or incorrect matching evaluating all the patient features. Of note, MTBs are often more inclined to refer patients to clinical trials in their own institution, even if not providing a matched therapy, rather than suggesting patients to move to other hospitals. Many digital tools have been developed to ease clinical trial access, indicating the most pertinent trials to each patient according to its clinical and genomic profiles. For instance, MatchMiner is an open-source software used at the Dana Farber Cancer Institute since 2017, and with October 2020 it has allowed for the enrolment of more than 118 cancer patients in a genomic-matched clinical trial [82]. MolecularMatch is another tool capable of matching patients’ characteristics with clinical trials and precision medicine indications, relying on a self-learning software [83]. Nevertheless, these systems can help, but not replace, the nowadays necessary systematic matching of patients performing genomic tests to clinical trials, and efficacious strategies should be developed to also guarantee access to studies for patients living in rural areas, as Canada is trying to institute with decentralized clinical trials [84].

The off-label use of drugs also represents a major problem and depends on the health system in which the MTB operates. In fact, in the USA it is easier to access off-label treatment and about 30% of prescriptions are ascribed to use this modality, while in Europe it is more difficult, and ad hoc clinical trials such as DRUP have been designed to address this issue [85]. A closer collaboration between Pharmaceutical Companies and National Healthcare Institutes has been demonstrated to be possible in The Netherlands and should be pursued also in other countries. The employment in MTBs of personnel dedicated to easing the access to drugs is another noticeable option successfully carried out in the I-PREDICT trial [55].

**Figure 1:**
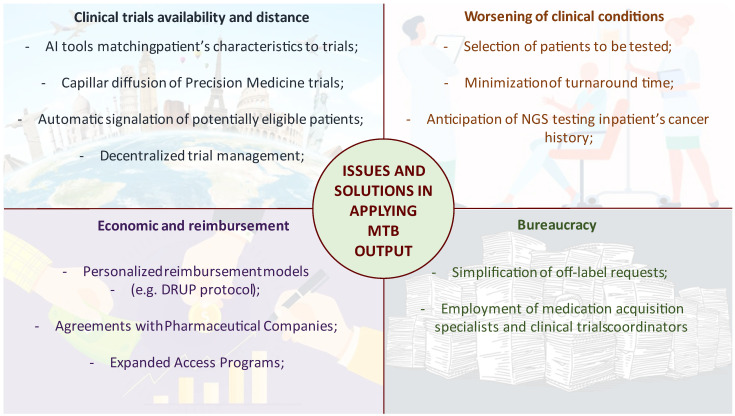
Schematization of the most frequent causes of MTB indication inapplicability and the proposed solutions.

Figure 1 exemplifies the most frequent issues that impede the administration of the therapies proposed during MTBs and the solutions that have been developed. 

## 6. Conclusions

The heterogeneity in reporting MTB results complicates trials’ comparisons. Hence, extensive efforts are required to standardize the interpretation of molecular alterations in terms of actionability, therapeutic indications, and the outcome data to be included in publications. Drug accessibility remains the most common reason impeding the reception of a molecular matched treatment, followed by worsening patient clinical conditions. An increased and systematic utilization of digital tools based on artificial intelligence is expected to help MTBs and clinicians to overcome these issues and to guarantee the most up-to-date treatment opportunities for cancer patients.

## References

[1] Goodwin S., McPherson J.D., McCombie W.R. (2016). Coming of age: Ten years of next-generation sequencing technologies. Nat. Rev. Genet..

[2] Janssens J., Gallagher W.M., Dean A., Ussia G., Stamp G. (2017). Tumor Profiling-Directed Precision Cancer Therapy—Comparison of Commercial and Academic Clinical Utility. Int. J. Surg. Surg. Proced..

[3] FoundationOne CDx|Foundation Medicine. https://www.foundationmedicine.com/test/foundationone-cdx.

[4] Woodhouse R., Li M., Hughes J., Delfosse D., Skoletsky J., Ma P., Meng W., Dewal N., Milbury C., Clark T. (2020). Clinical and analytical validation of FoundationOne Liquid CDx, a novel 324-Gene cfDNA-based comprehensive genomic profiling assay for cancers of solid tumor origin. PLoS ONE.

[5] Carter P., Alifrangis C., Cereser B., Chandrasinghe P., Belluz L.D.B., Herzog T., Levitan J., Moderau N., Schwartzberg L., Tabassum N. (2018). Does molecular profiling of tumors using the Caris molecular intelligence platform improve outcomes for cancer patients?. Oncotarget.

[6] Rolfo C., Manca P., Salgado R., Van Dam P., Dendooven A., Gandia J.F., Rutten A., Lybaert W., Vermeij J., Gevaert T. (2018). Multidisciplinary molecular tumour board: A tool to improve clinical practice and selection accrual for clinical trials in patients with cancer. ESMO Open.

[7] Luchini C., Lawlor R.T., Milella M., Scarpa A. (2020). Molecular Tumor Boards in Clinical Practice. Trends Cancer.

[8] Landrum M.J., Chitipiralla S., Brown G.R., Chen C., Gu B., Hart J., Hoffman D., Jang W., Kaur K., Liu C. (2020). ClinVar: Improvements to accessing data. Nucleic Acids Res..

[9] Tate J.G., Bamford S., Jubb H.C., Sondka Z., Beare D.M., Bindal N., Boutselakis H., Cole C.G., Creatore C., Dawson E. (2019). COSMIC: The Catalogue of Somatic Mutations in Cancer. Nucleic Acids Res..

[10] Chakravarty D., Gao J., Phillips S., Kundra R., Zhang H., Wang J., Rudolph J.E., Yaeger R., Soumerai T., Nissan M.H. (2017). OncoKB: A Precision Oncology Knowledge Base. JCO Precis. Oncol..

[11] FDA FDA Recognizes Memorial Sloan-Kettering Database of Molecular Tumor Marker Information. https://www.fda.gov/drugs/resources-information-approved-drugs/fda-recognizes-memorial-sloan-kettering-database-molecular-tumor-marker-information.

[12] Marabelle A., Fakih M., Lopez J., Shah M., Shapira-Frommer R., Nakagawa K., Chung H.C., Kindler H.L., Lopez-Martin J.A., Miller W.H. (2020). Association of tumour mutational burden with outcomes in patients with advanced solid tumours treated with pembrolizumab: Prospective biomarker analysis of the multicohort, open-label, phase 2 KEYNOTE-158 study. Lancet Oncol..

[13] FDA FDA Grants Accelerated Approval to Dostarlimab-Gxly for dMMR Advanced Solid Tumors. https://www.fda.gov/drugs/resources-information-approved-drugs/fda-grants-accelerated-approval-dostarlimab-gxly-dmmr-advanced-solid-tumors.

[14] Mateo J., Chakravarty D., Dienstmann R., Jezdic S., Gonzalez-Perez A., Lopez-Bigas N., Ng C., Bedard P., Tortora G., Douillard J.-Y. (2018). A framework to rank genomic alterations as targets for cancer precision medicine: The ESMO Scale for Clinical Actionability of molecular Targets (ESCAT). Ann. Oncol..

[15] Li M.M., Datto M., Duncavage E.J., Kulkarni S., Lindeman N.I., Roy S., Tsimberidou A.M., Vnencak-Jones C.L., Wolff D.J., Younes A. (2017). Standards and Guidelines for the Interpretation and Reporting of Sequence Variants in Cancer: A Joint Consensus Recommendation of the Association for Molecular Pathology, American Society of Clinical Oncology, and College of American Pathologists. J. Mol. Diagn..

[16] Eisenhauer E.A., Therasse P., Bogaerts J., Schwartz L.H., Sargent D., Ford R., Dancey J., Arbuck S., Gwyther S., Mooney M. (2009). New response evaluation criteria in solid tumours: Revised RECIST guideline (version 1.1). Eur. J. Cancer.

[17] Kato S., Kim K.H., Lim H.J., Boichard A., Nikanjam M., Weihe E., Kuo D.J., Eskander R.N., Goodman A., Galanina N. (2020). Real-world data from a molecular tumor board demonstrates improved outcomes with a precision N-of-One strategy. Nat. Commun..

[18] Basse C., Morel C., Alt M., Sablin M.P., Franck C., Pierron G., Callens C., Melaabi S., Masliah-Planchon J., Bataillon G. (2018). Relevance of a molecular tumour board (MTB) for patients’ enrolment in clinical trials: Experience of the Institut Curie. ESMO Open.

[19] Le Tourneau C., Delord J.-P., Gonçalves A., Gavoille C., Dubot C., Isambert N., Campone M., Trédan O., Massiani M.-A., Mauborgne C. (2015). Molecularly targeted therapy based on tumour molecular profiling versus conventional therapy for advanced cancer (SHIVA): A multicentre, open-label, proof-of-concept, randomised, controlled phase 2 trial. Lancet Oncol..

[20] Larson K.L., Huang B., Weiss H.L., Hull P., Westgate P.M., Miller R.W., Arnold S.M., Kolesar J.M. (2021). Clinical Outcomes of Molecular Tumor Boards: A Systematic Review. JCO Precis. Oncol..

[21] Weiss G., Hoff B., Whitehead R., Sangal A., Gingrich S., Penny R., Mallery D., Morris S., Thompson E., Loesch D. (2015). Evaluation and comparison of two commercially available targeted next-generation sequencing platforms to assist oncology decision making. OncoTargets Ther..

[22] Pishvaian M.J., Blais E.M., Bender R.J., Rao S., Boca S.M., Chung V., E Hendifar A., Mikhail S., Sohal D.P.S., Pohlmann P.R. (2019). A virtual molecular tumor board to improve efficiency and scalability of delivering precision oncology to physicians and their patients. JAMIA Open.

[23] Hoefflin R., Geißler A.-L., Fritsch R., Claus R., Wehrle J., Metzger P., Reiser M., Mehmed L., Fauth L., Heiland D.H. (2018). Personalized Clinical Decision Making Through Implementation of a Molecular Tumor Board: A German Single-Center Experience. JCO Precis. Oncol..

[24] Horak P., Klink B., Heining C., Gröschel S., Hutter B., Fröhlich M., Uhrig S., Hübschmann D., Schlesner M., Eils R. (2017). Precision oncology based on omics data: The NCT Heidelberg experience. Int. J. Cancer.

[25] Beltran H., Eng K., Mosquera J.M., Sigaras A., Romanel A., Rennert H., Kossai M., Pauli C., Faltas B., Fontugne J. (2015). Whole-Exome Sequencing of Metastatic Cancer and Biomarkers of Treatment Response. JAMA Oncol..

[26] Mosele F., Remon J., Mateo J., Westphalen C., Barlesi F., Lolkema M., Normanno N., Scarpa A., Robson M., Meric-Bernstam F. (2020). Recommendations for the use of next-generation sequencing (NGS) for patients with metastatic cancers: A report from the ESMO Precision Medicine Working Group. Ann. Oncol..

[27] Pagès A., Foulon S., Zou Z., Lacroix L., Lemare F., de Baère T., Massard C., Soria J.-C., Bonastre J. (2017). The cost of molecular-guided therapy in oncology: A prospective cost study alongside the MOSCATO trial. Genet. Med..

[28] Galsky M.D., Stensland K.D., McBride R.B., Latif A., Moshier E., Oh W.K., Wisnivesky J. (2015). Geographic Accessibility to Clinical Trials for Advanced Cancer in the United States. JAMA Intern. Med..

[29] Li N., Huang H.-Y., Wu D.-W., Yang Z.-M., Wang J., Wang J.-S., Wang S.-H., Fang H., Yu Y., Bai Y. (2019). Changes in clinical trials of cancer drugs in mainland China over the decade 2009–18: A systematic review. Lancet Oncol..

[30] Chakraborty S., Mallick I., Luu H.N., Bhattacharyya T., Arunsingh M., Achari R.B., Chatterjee S. (2021). Geographic disparities in access to cancer clinical trials in India. Ecancermedicalscience.

[31] Meropol N.J., Buzaglo J.S., Millard J., Damjanov N., Miller S.M., Ridgway C., Ross E.A., Sprandio J.D., Watts P. (2007). Barriers to Clinical Trial Participation as Perceived by Oncologists and Patients. J. Natl. Compr. Cancer Netw..

[32] Pantziarka P., Capistrano R.I., De Potter A., Vandeborne L., Bouche G. (2021). An Open Access Database of Licensed Cancer Drugs. Front. Pharmacol..

[33] Collyar D.E. (2020). Time to Treat Financial Toxicity for Patients. Cancer J..

[34] Meyers D.E., Meyers B.S., Msc T.M.C., Wright K., Gyawali B., Prasad V., Sullivan R., Booth C.M. (2021). Trends in drug revenue among major pharmaceutical companies: A 2010-2019 cohort study. Cancer.

[35] Moore T.J., Heyward J., Anderson G., Alexander G.C. (2020). Variation in the estimated costs of pivotal clinical benefit trials supporting the US approval of new therapeutic agents, 2015–2017: A cross-sectional study. BMJ Open.

[36] Unger J.M., Cook E., Tai E., Bleyer A. (2016). The Role of Clinical Trial Participation in Cancer Research: Barriers, Evidence, and Strategies. Am. Soc. Clin. Oncol. Educ. Book.

[37] Mattson M.E., Curb J., McArdle R. (1985). Participation in a clinical trial: The patients’ point of view. Control. Clin. Trials.

[38] Reddy N., Subbiah V. (2021). Right to Try, expanded access use, Project Facilitate, and clinical trial reform. Ann. Oncol..

[39] Stout J., Smith C., Buckner J., Adjei A.A., Wentworth M., Tilburt J.C., Master Z. (2021). Oncologists’ reflections on patient rights and access to compassionate use drugs: A qualitative interview study from an academic cancer center. PLoS ONE.

[40] Dalton W., Forde P.M., Kang H., Connolly R.M., Stearns V., Gocke C.D., Eshleman J.R., Axilbund J., Petry D., Geoghegan C. (2017). Personalized Medicine in the Oncology Clinic: Implementation and Outcomes of the Johns Hopkins Molecular Tumor Board. JCO Precis. Oncol..

[41] Hirshfield K.M., Tolkunov D., Zhong H., Ali S.M., Stein M.N., Murphy S., Vig H., Vazquez A., Glod J., Moss R.A. (2016). Clinical Actionability of Comprehensive Genomic Profiling for Management of Rare or Refractory Cancers. Oncologist.

[42] Harada S., Arend R., Dai Q., Levesque J.A., Winokur T.S., Guo R., Heslin M.J., Nabell L., Nabors L.B., Limdi N.A. (2017). Implementation and utilization of the molecular tumor board to guide precision medicine. Oncotarget.

[43] Moore D.A., Kushnir M., Mak G., Winter H., Curiel T., Voskoboynik M., Moschetta M., Rozumna-Martynyuk N., Balbi K., Bennett P. (2019). Prospective analysis of 895 patients on a UK Genomics Review Board. ESMO Open.

[44] Von Hoff D.D., Stephenson J.J., Rosen P., Loesch D.M., Borad M.J., Anthony S., Jameson G.S., Brown S., Cantafio N., Richards D.A. (2010). Pilot Study Using Molecular Profiling of Patients’ Tumors to Find Potential Targets and Select Treatments for Their Refractory Cancers. J. Clin. Oncol..

[45] Cobain E.F., Wu Y.-M., Vats P., Chugh R., Worden F., Smith D.C., Schuetze S.M., Zalupski M.M., Sahai V., Alva A. (2021). Assessment of Clinical Benefit of Integrative Genomic Profiling in Advanced Solid Tumors. JAMA Oncol..

[46] Meric-Bernstam F., Brusco L., Shaw K., Horombe C., Kopetz S., Davies M.A., Routbort M.J., Piha-Paul S., Janku F., Ueno N.T. (2015). Feasibility of Large-Scale Genomic Testing to Facilitate Enrollment Onto Genomically Matched Clinical Trials. J. Clin. Oncol..

[47] Stockley T., Oza A., Berman H.K., Leighl N.B., Knox J.J., Shepherd F.A., Chen E.X., Krzyzanowska M., Dhani N., Joshua A. (2016). Molecular profiling of advanced solid tumors and patient outcomes with genotype-matched clinical trials: The Princess Margaret IMPACT/COMPACT trial. Genome Med..

[48] Zehir A., Benayed R., Shah R.H., Syed A., Middha S., Kim H.R., Srinivasan P., Gao J., Chakravarty D., Devlin S.M. (2017). Mutational landscape of metastatic cancer revealed from prospective clinical sequencing of 10,000 patients. Nat. Med..

[49] Eubank M.H., Hyman D.M., Kanakamedala A.D., Gardos S., Wills J.M., Stetson P.D. (2016). Automated eligibility screening and monitoring for genotype-driven precision oncology trials. J. Am. Med Informatics Assoc..

[50] Tuxen I.V., Rohrberg K.S., Oestrup O., Ahlborn L.B., Schmidt A.Y., Spanggaard I., Hasselby J.P., Santoni-Rugiu E., Yde C.W., Mau-Sørensen M. (2019). Copenhagen prospective personalized oncology (COPPO)—Clinical utility of using molecu-lar profiling to select patients to phase I trials. Clin. Cancer Res..

[51] Radovich M., Kiel P.J., Nance S.M., Niland E.E., Parsley M.E., Ferguson M.E., Jiang G., Ammakkanavar N.R., Einhorn L.H., Cheng L. (2016). Clinical benefit of a precision medicine based approach for guiding treatment of refractory cancers. Oncotarget.

[52] Wheler J.J., Janku F., Naing A., Li Y., Stephen B., Zinner R., Subbiah V., Fu S., Karp D., Falchook G.S. (2016). Cancer Therapy Directed by Comprehensive Genomic Profiling: A Single Center Study. Cancer Res..

[53] Horak P., Heining C., Kreutzfeldt S., Hutter B., Mock A., Hüllein J., Fröhlich M., Uhrig S., Jahn A., Rump A. (2021). Com-prehensive genomic and transcriptomic analysis for guiding therapeutic decisions in patients with rare cancers. Cancer Discov..

[54] Rodon J., Soria J.-C., Berger R., Miller W.H., Rubin E., Kugel A., Tsimberidou A., Saintigny P., Ackerstein A., Braña I. (2019). Genomic and transcriptomic profiling expands precision cancer medicine: The WINTHER trial. Nat. Med..

[55] Sicklick J.K., Kato S., Okamura R., Schwaederle M., Hahn M.E., Williams C.B., De P., Krie A., Piccioni D.E., Miller V.A. (2019). Molecular profiling of cancer patients enables personalized combination therapy: The I-PREDICT study. Nat. Med..

[56] Rothwell D., Ayub M., Cook N., Thistlethwaite F., Carter L., Dean E., Smith N., Villa S., Dransfield J., Clipson A. (2019). Utility of ctDNA to support patient selection for early phase clinical trials: The TARGET study. Nat. Med..

[57] Nakamura Y., Taniguchi H., Ikeda M., Bando H., Kato K., Morizane C., Esaki T., Komatsu Y., Kawamoto Y., Takahashi N. (2020). Clinical utility of circulating tumor DNA sequencing in advanced gastrointestinal cancer: SCRUM-Japan GI-SCREEN and GOZILA studies. Nat. Med..

[58] Aftimos P., Oliveira M., Irrthum A., Fumagalli D., Sotiriou C., Gal-Yam E.N., Robson M.E., Ndozeng J., di Leo A., Ciruelos E.M. (2011). Genomic and transcriptomic analyses of breast cancer primaries and matched metastases in Aurora, the breast international group (Big) molecular screening initiative. Cancer Discov..

[59] Massard C., Michiels S., Ferté C., Le Deley M.-C., Lacroix L., Hollebecque A., Verlingue L., Ileana E., Rosellini S., Ammari S. (2017). High-Throughput Genomics and Clinical Outcome in Hard-to-Treat Advanced Cancers: Results of the MOSCATO 01 Trial. Cancer Discov..

[60] Flaherty K.T., Gray R., Chen A., Li S., Patton D., Hamilton S.R., Williams P.M., Mitchell E.P., Iafrate A.J., Sklar J. (2020). The Molecular Analysis for Therapy Choice (NCI-MATCH) Trial: Lessons for Genomic Trial Design. JNCI J. Natl. Cancer Inst..

[61] Trédan O., Wang Q., Pissaloux D., Cassier P., de la Fouchardière A., Fayette J., Desseigne F., Ray-Coquard I., de la Fouchardiere C., Frappaz D. (2019). Molecular screening program to select molecular-based recommended therapies for metastatic cancer patients: Analysis from the ProfiLER trial. Ann. Oncol..

[62] Varnier R., Le Saux O., Chabaud S., Garin G., Sohier E., Wang Q., Paindavoine S., Pérol D., Baudet C., Attignon V. (2019). Actionable molecular alterations in advanced gynaecologic malignancies: Updated results from the ProfiLER programme. Eur. J. Cancer.

[63] Powles T., Carroll D., Chowdhury S., Gravis G., Joly F., Carles J., Fléchon A., Maroto P., Petrylak D., Rolland F. (2021). An adaptive, biomarker-directed platform study of durvalumab in combination with targeted therapies in advanced urothelial cancer. Nat. Med..

[64] Hainsworth J.D., Meric-Bernstam F., Swanton C., Hurwitz H., Spigel D.R., Sweeney C., Burris H.A., Bose R., Yoo B., Stein A. (2018). Targeted Therapy for Advanced Solid Tumors on the Basis of Molecular Profiles: Results from MyPathway, an Open-Label, Phase IIa Multiple Basket Study. J. Clin. Oncol..

[65] Friedman C.F., Hainsworth J.D., Kurzrock R., Spigel D.R., Burris H.A., Sweeney C.J., Meric-Bernstam F., Wang Y., Levy J., Grindheim J. (2022). Atezolizumab Treatment of Tumors with High Tumor Mutational Burden from MyPathway, a Multicenter, Open-Label, Phase IIa Multiple Basket Study. Cancer Discov..

[66] Meric-Bernstam F., Hurwitz H., Raghav K.P.S., McWilliams R.R., Fakih M., VanderWalde A., Swanton C., Kurzrock R., Burris H., Sweeney C. (2019). Pertuzumab plus trastuzumab for HER2-amplified metastatic colorectal cancer (MyPathway): An updated report from a multicentre, open-label, phase 2a, multiple basket study. Lancet Oncol..

[67] Javle M., Borad M.J., Azad N.S., Kurzrock R., Abou-Alfa G.K., George B., Hainsworth J., Meric-Bernstam F., Swanton C., Sweeney C.J. (2021). Pertuzumab and trastuzumab for HER2-positive, metastatic biliary tract cancer (MyPathway): A multicentre, open-label, phase 2a, multiple basket study. Lancet Oncol..

[68] Kurzrock R., Bowles D., Kang H., Meric-Bernstam F., Hainsworth J., Spigel D., Bose R., Burris H., Sweeney C., Beattie M. (2020). Targeted therapy for advanced salivary gland carcinoma based on molecular profiling: Results from MyPathway, a phase IIa multiple basket study. Ann. Oncol..

[69] Mangat P.K., Halabi S., Bruinooge S.S., Garrett-Mayer E., Alva A., Janeway K.A., Stella P.J., Voest E., Yost K.J., Perlmutter J. (2018). Rationale and Design of the Targeted Agent and Profiling Utilization Registry Study. JCO Precis. Oncol..

[70] Al Baghdadi T., Garrett-Mayer E., Halabi S., Mangat P.K., Rich P., Ahn E.R., Chai S., Rygiel A.L., Osayameh O., Antonelli K.R. (2020). Sunitinib in Patients with Metastatic Colorectal Cancer (mCRC) with FLT-3 Amplification: Results from the Targeted Agent and Profiling Utilization Registry (TAPUR) Study. Target. Oncol..

[71] Fisher J.G., Tait D., Garrett-Mayer E., Halabi S., Mangat P.K., Schink J.C., Alvarez R.H., Veljovich D., Cannon T.L., Crilley P.A. (2020). Cetuximab in Patients with Breast Cancer, Non-Small Cell Lung Cancer, and Ovarian Cancer Without KRAS, NRAS, or BRAF Mutations: Results from the Targeted Agent and Profiling Utilization Registry (TAPUR) Study. Target. Oncol..

[72] Ahn E.R., Mangat P.K., Garrett-Mayer E., Halabi S., Dib E.G., Haggstrom D.E., Alguire K.B., Calfa C.J., Cannon T.L., Crilley P.A. (2020). Palbociclib in Patients With Non–Small-Cell Lung Cancer With *CDKN2A* Alterations: Results From the Targeted Agent and Profiling Utilization Registry Study. JCO Precis. Oncol..

[73] Al Baghdadi T., Halabi S., Garrett-Mayer E., Mangat P.K., Ahn E.R., Sahai V., Alvarez R.H., Kim E.S., Yost K.J., Rygiel A.L. (2019). Palbociclib in Patients with Pancreatic and Biliary Cancer With *CDKN2A* Alterations: Results From the Targeted Agent and Profiling Utilization Registry Study. JCO Precis. Oncol..

[74] Lee J., Kim S.T., Kim K., Lee H., Kozarewa I., Mortimer P.G., Odegaard J.I., Harrington E.A., Lee J., Lee T. (2019). Tumor Genomic Profiling Guides Patients with Metastatic Gastric Cancer to Targeted Treatment: The VIKTORY Umbrella Trial. Cancer Discov..

[75] Park K.H., Choi J.Y., Lim A.-R., Kim J.W., Choi Y.J., Lee S., Sung J.S., Chung H.-J., Jang B., Yoon D. (2021). Genomic Landscape and Clinical Utility in Korean Advanced Pan-Cancer Patients from Prospective Clinical Sequencing: K-MASTER Program. Cancer Discov..

[76] Van Der Velden D.L., Hoes L.R., Van Der Wijngaart H., van Berge Henegouwen J.M., Van Werkhoven E., Roepman P., Schilsky R.L., De Leng W.W.J., Huitema A.D.R., Nuijen B. (2019). The Drug Rediscovery protocol facilitates the expanded use of existing anticancer drugs. Nature.

[77] Doorn-Khosrovani S.V.W.V., Roy A.P.-V., Van Saase L., Van Der Graaff M., Gijzen J., Sleijfer S., Hoes L., Henegouwen J.V.B., Van Der Wijngaart H., Van Der Velden D. (2019). Personalised reimbursement: A risk-sharing model for biomarker-driven treatment of rare subgroups of cancer patients. Ann. Oncol..

[78] Hoes L.R., van Berge Henegouwen J.M., van der Wijngaart H., Zeverijn L.J., van der Velden D.L., van de Haar J., Roepman P., de Leng W.J., Jansen A.M., van Werkhoven E. (2022). Patients with Rare Cancers in the Drug Rediscovery Protocol (DRUP) Benefit from Genomics-Guided Treatment. Clin. Cancer Res..

[79] Biswas D., Ganeshalingam J., Wan J.C.M. (2020). The future of liquid biopsy. Lancet Oncol..

[80] Turner N.C., Kingston B., Kilburn L.S., Kernaghan S., Wardley A.M., Macpherson I.R., Baird R.D., Roylance R., Stephens P., Oikonomidou O. (2020). Circulating tumour DNA analysis to direct therapy in advanced breast cancer (plasmaMATCH): A multicentre, multicohort, phase 2a, platform trial. Lancet Oncol..

[81] Cherny N.I., Dafni U., Bogaerts J., Latino N.J., Pentheroudakis G., Douillard J.-Y., Tabernero J., Zielinski C., Piccart M.J., de Vries E.G.E. (2017). ESMO-Magnitude of Clinical Benefit Scale version 1.1. Ann. Oncol..

[82] Mazor T., Kumari P., Lindsay J., Ovalle A., Siegel E., Yu J., Hassett M., Cerami E. (2020). MatchMiner: Computational matching of cancer patients to precision medicine clinical trials. Eur. J. Cancer.

[83] MMPower|Molecular Knowledge Base Platform|MolecularMatch. https://www.molecularmatch.com/mmpower/.

[84] Sundquist S., Batist G., Brodeur-Robb K., Dyck K., Eigl B.J., Lee D.K., Limoges J., Longstaff H., Pankovich J., Sadura A. (2021). CRAFT—A Proposed Framework for Decentralized Clinical Trials Participation in Canada. Curr. Oncol..

[85] De Vries E., Cherny N., Voest E. (2019). When is off-label off-road?. Ann. Oncol..

